# Player ranking is associated with physical performance and body composition in 12-year-old female soccer players

**DOI:** 10.3389/fspor.2026.1823705

**Published:** 2026-07-23

**Authors:** Sigurdur Benediktsson, Elvar S. Saevarsson, Runa Stefansdottir, Erlingur Johannsson, Vaka Rognvaldsdottir

**Affiliations:** 1 Center of Sport and Health Sciences, School of Education, University of Iceland, Reykjavik, Iceland; 2 Department of Sport and Physical Activity, Western Norway University of Applied Sciences, Bergen, Norway

**Keywords:** body composition, female, maturity, physical performance, youth soccer

## Abstract

**Background:**

The relative contribution of biological maturation and body composition to performance in youth female soccer remains unclear. This study compared skeletal maturity, body composition, and physical performance across ranking groups in 12-year-old female players within a community club system.

**Methods:**

Eighty-eight players (11.9 ± 0.3 years) completed bone age (BA) assessment from non-dominant wrist radiographs, DXA body composition testing, and field-based measures including countermovement jump (CMJ), Illinois change-of-direction test with ball (ICODT-BALL), 40-m sprint, and Yo-Yo intermittent recovery test level 1 (IR1). Players were assigned by their coaches to three performance-based ranking groups. Between-group differences were analysed using one-way ANOVA with *η*^2^ effect sizes and Holm-adjusted *post-hoc* comparisons.

**Results:**

BA did not differ significantly between ranking groups (*p* = 0.590). Players in Rank 1 (highest rank) showed lower body fat percentage (Fat%) (*p* = 0.015), whereas no significant differences were found in fat-free mass index (FFMI). Clear performance differences were observed as Rank 1 demonstrated higher CMJ height (*p* < 0.001), faster ICODT-BALL times (*p* < 0.001), and faster 40-m sprint performance (*p* < 0.001). No significant differences were found for the IR1 test between ranking groups (*p* = 0.360).

**Conclusion:**

Performance ranking at chronological age 12 primarily reflects agility performance with the ball, 40-m sprint performance and CMJ height, but not biological bone age. Higher-ranked players also demonstrated lower Fat%, whereas FFMI did not differ significantly between ranking groups.

## 1 Introduction

Talent identification in youth sport is a multidimensional process shaped by the interaction of physical, technical, psychological, and developmental factors, making long-term predictions inherently uncertain (1, 2). Methodological inconsistency and variation in testing approaches further limit the reliability and practical application of current evidence (3). Although research interest has increased, studies focusing specifically on youth female soccer players remain limited, with much of the literature derived from male or mixed-sex samples (4, 5). Therefore, developmental and maturation-related factors specific to girls are still not well understood within talent development pathways (6, 7). Talent identification at early ages may favour early biological maturation and overlook later developers. Understanding how maturity and physical performance relate to soccer talent potential is important for fair selection and long-term development in female youth soccer (8, 9). Adolescence is a critical period in which biological maturation drives substantial changes in growth, body composition, coordination, and strength (10). Girls typically enter puberty earlier than boys, which accelerates the tempo of growth and alters neuromuscular function (11, 12). Research in female soccer indicates that maturation-related differences are often expressed through body size and composition, while evidence linking biological maturity directly to performance differentiation remains limited (13).

In female youth soccer, sprint speed, explosive power, and change-of-direction ability have been identified as key physical characteristics associated with progression through competitive levels (14). Emerging evidence further suggests that agility tests incorporating ball control may differentiate competitive level more effectively than change-of-direction tasks performed without the ball (15). Given the central role of these high-intensity performance capacities in early talent identification, an important question is whether such differences reflect true functional ability or underlying biological maturity.

Biological maturity reflects skeletal development rather than time since birth and is typically assessed in terms of bone age (BA), measured from hand-wrist radiographs. In contrast, chronological age represents years lived and does not capture individual variation in growth tempo. In boys, advanced biological maturity, indicated by higher BA relative to chronological age, is consistently associated with superior speed, jumping, and strength, increasing the likelihood of selection into youth academies (11, 16–18). In girls, associations between biological maturity and physical performance appear weaker and less consistent, likely reflecting sex-specific patterns of growth and body composition during puberty. Compared with boys, girls gain proportionally more fat mass and less lean mass during puberty, which may help explain weaker links between maturity and performance (19, 20). Similarly, BA is positively associated with performance in adolescent boys but shows limited or inconsistent associations in girls aged 13–15 years, likely because most girls have passed peak height velocity by this stage, reducing the variance in biological maturity across peers (21).

In research on youth female soccer players, maturity is most often estimated indirectly using anthropometric models such as years from peak height velocity (PHV). Although widely used, the PHV offset shows limited accuracy in girls, with systematic bias around PHV and substantial individual error (22, 23). Direct BA measurement from hand-wrist x-rays is the most accurate method of quantifying biological maturity (24, 25). When combined with dual-energy x-ray absorptiometry (DXA), precise measures of fat mass and fat-free mass can be obtained, enabling clear distinction between maturity and body composition, both of which are relevant to sprint, jump, and change-of-direction performance in early adolescent girls (26). However, few studies in female soccer have applied both direct BA assessment and DXA-derived body composition within the same cohort (19), most investigating these factors independently (27).

Body composition, particularly fat mass and body fat percentage (Fat%) has shown stronger associations with sprint and jump performance than BA in 12-year-old female soccer players (19). This suggests that performance differences in early adolescent girls may align more closely with body composition than with BA at this age. However, no studies have specifically examined whether differences in biological maturity and body composition are reflected in performance ranking within community-based girls' soccer, where selection decisions are typically based on observed performance during regular training and match play, rather than on formal academy evaluation processes. Therefore, the aim of this study was to examine differences in BA, body composition, and physical performance across ranking groups in 12-year-old female soccer players.

## 2 Materials and methods

### 2.1 Study design and participants

**Figure 1:**
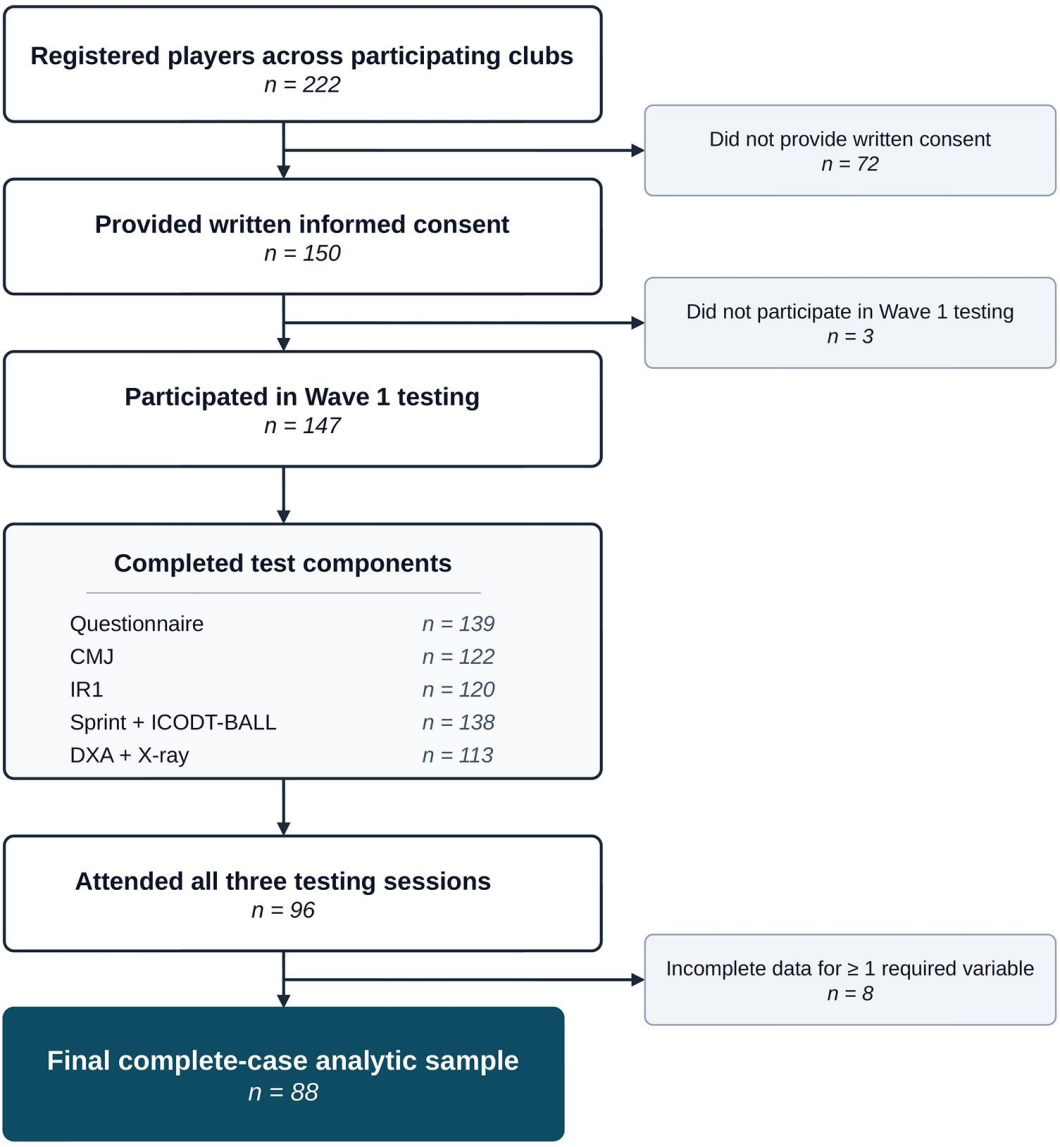
Participant selection flowchart.

This cross-sectional study used baseline data from the ongoing longitudinal SKORA (Soccer Knowledge for Optimal Resilience and Athletic Performance in Female Youth) project, which investigates growth, maturation, and performance development in young female soccer players in Iceland. The present analysis examined the associations between BA, DXA-derived body composition, and physical performance in 12-year-old female soccer players. Eligible participants were girls born in 2012 and registered with community-based soccer clubs in the Capital Region of Iceland. Twelve clubs were invited to participate, of which ten agreed, providing broad coverage of the local youth female soccer environment. At the time of recruitment, 222 players were registered across these clubs and 150 provided written informed consent. Inclusion criteria required both regular participation in team training and being free from any acute injury or medical conditions that could affect safe completion of testing. Team training exposure ranged from three to four sessions per week, with each session lasting 60–90 min. Testing was conducted across three sessions during Wave 1 data collection. Of the 150 players who consented, 147 participated in Wave 1 testing and 96 attended all three testing sessions. The remaining 59 Wave 1 participants were excluded from the final analysis due to data loss attributable to technical failure or incomplete data capture across one or more required test components. Complete valid data for BA, body composition and all required physical performance measures were available for 88 players, who comprised the final analytic sample. The participant selection flowchart is presented in Figure 1.

### 2.2 Data collection

The study was conducted during spring 2024. Testing sessions were scheduled during regular team trainings to minimise disruption. Each club completed two testing days, with additional sessions provided for larger groups if needed. All field-based testing was conducted at each club's training facility. A standardised warm-up was conducted before each performance test, as appropriate. The first data collection day included questionnaire administration, countermovement jump (CMJ), and the Yo-Yo intermittent recovery test level 1 (IR1). The second testing day included the Illinois change-of-direction test with ball (ICODT-BALL) and the 40-m sprint test. Body composition and BA were assessed separately using DXA and x-ray at the Icelandic Heart Association between May 23 and June 7, 2024, approximately 1–8 weeks after field testing due to scanner availability.

### 2.3 Questionnaire

Participants completed a standardised digital questionnaire at their team facilities to collect background and training history information. As part of the participant training history, subjects were asked to report the age at which they began organised soccer participation (“How old were you when you started playing soccer with a sports team?”). Response categories ranged from 4 years old and younger to 12 years of age.

### 2.4 Performance ranking

Coaches were asked to evaluate and rank each player's current soccer performance-related qualities using a three-point scale, where Rank 1 represented the highest-performing group. A player in Rank 1 usually played with the first (best) team or had the ability to do so, a player in Rank 2 with the second team or had the ability to do so and a player in Rank 3 with the third team or had not yet been assigned to a team. All rankings were made independently by each player's own team head coach, who was familiar with the players' performance throughout the season. The ranking was based on the coach's overall evaluation of current soccer performance within the team context, rather than on separate written sub criteria for technical, tactical, physical or psychological qualities. Therefore, the rank variable should be interpreted as a coach-derived overall performance classification, reflecting the coach's season-long impression of each player's ability and team placement.

Formal inter-rater reliability was not assessed and no shared evaluation sessions were conducted across teams. However, the ranking categories were defined in advance and rankings were internally consistent within each club. Accordingly, the rank variable reflects coach evaluation within the specific team context rather than a standardised multidimensional talent assessment across clubs.

### 2.5 Ethical considerations

Ethical approval for the study was granted by the Icelandic National Bioethics Committee and the Icelandic Data Protection Authority (study number VSN-24-023). All procedures adhered to the principles outlined in the Declaration of Helsinki. Written informed consent to participate in this study was obtained from the participants and their legal guardians.

### 2.6 Anthropometric and body composition assessment

Body composition was assessed at the Icelandic Heart Association using a GE Lunar iDXA Pro Advance dual-energy x-ray absorptiometry (DXA) scanner (GE HealthCare, Chicago, IL, USA), operated by a certified radiographer. All assessments were conducted in the afternoon. Body weight was recorded to the nearest 0.1 kg using a digital scale (Seca 813, Seca Ltd., Birmingham, UK), with players wearing light indoor clothing and no shoes. Standing height was measured to the nearest 0.1 cm using a calibrated stadiometer (Seca 123, Seca Ltd., Birmingham, UK), with participants barefoot. Body mass index (BMI) was calculated as weight in kilograms divided by height in metres squared (kg/m^2^). From the DXA scan, body fat percentage (Fat%) was calculated as total fat mass divided by total body mass, multiplied by 100. Fat-free mass (FFM) was derived by subtracting total fat mass from total body mass in kilograms. Fat-free mass index (FFMI) was calculated as fat-free mass in kilograms divided by height in metres squared (kg/m^2^) (26, 28, 29).

### 2.7 Bone age (BA)

Biological maturity was assessed using BA estimation from a left-hand wrist x-ray. All radiographs were obtained at the Icelandic Heart Association using a Siemens Ysio Max system with a FLUOROSPOT Compact imaging unit (software version VE10; Siemens Healthineers). The imaging field included the entire hand and approximately 3 cm of the distal radius and ulna to ensure adequate visualisation of the epiphyseal growth plates. Standardised acquisition parameters were used, including a tube-detector distance of 1 m, an x-ray tube voltage of 50 kV, and exposure settings of 1–1.5 mAs. No image filtering or post-processing procedures were applied. Radiographic images were analysed using BoneXpert software (stand-alone version 3.4.1.0; Visiana, Holte, Denmark), which automatically generated eight to thirteen independent BA estimates based on the Greulich–Pyle reference atlas (30). The use of automated analysis eliminated inter- and intra-observer variability, enhancing reliability. Validation studies indicate high accuracy and consistency with the method (31).

### 2.8 Countermovement jump (CMJ)

The CMJ test was used to evaluate vertical lower body jump height. Following two to three supervised practice attempts, players performed three maximal jumps with hands placed on their hips to eliminate arm-swing contribution. From an upright stance, players were instructed to jump as high as possible after an initial knee flexion. They were also instructed to straighten their legs in the air. Jumps were recorded using a ForceDecks FDmini V.2 platform (VALD, Brisbane, Australia). Jump height was calculated using the impulse-momentum method, which estimates height based on impulse metrics prior to take-off (32). Peak power was normalised to body mass (W·kg−1), and the best of the three attempts was retained for analysis. Players were asked to land with their feet straight.

### 2.9 Yo-yo intermittent recovery test level 1 (IR1)

Intermittent endurance capacity was assessed using the IR1 test, following the original protocols (33, 34). Participants completed a standardised 20-minute warmup involving ten minutes of low-intensity running, 5 min of dynamic stretching, and three progressive 40–50 m linear sprints. The IR1 test involved repeated 2 × 20-m shuttle runs at incrementally increasing speeds, combined with 10 s of active recovery. The initial running speed was set at 10.0 km·h^−1^, with subsequent speed increments according to a standardised audio signal to ensure consistent pacing for the participants. The test was stopped after two unsuccessful attempts to maintain the required running speed. Total distance covered (in meters) was recorded for analysis.

### 2.10 40-m sprint

Sprint performance was assessed using a 40-m linear sprint test (40-m sprint) on an artificial turf. After a standardised 20-minute warmup (10 min of easy running, 5 min of dynamic stretching, three progressive accelerations, and two maximal 20-m sprints), the participants completed three maximal sprints with 2–3 min of passive recovery between attempts. The starting position was a standing split stance, with the front foot placed 60 cm behind the first photogate. Sprint times were recorded using a portable Witty photogate system (Microgate, Bolzano, Italy). The initial gate was positioned 50 cm above the track, and gates at 20, 30, and 40 m were set at a height of 120 cm (35). The fastest time across the three trials was used for analysis (35).

### 2.11 Illinois change-of-direction test with ball

Change-of-direction performance with ball control was assessed using an Illinois change-of-direction test with ball (ICODT-BALL) to include dribbling. The modified test has demonstrated acceptable reliability and construct validity in youth soccer populations (36). The course measured 10 m in length and 5 m in width and was set up according to standard test dimensions (37, 38). Two identical size 4 (Select) soccer balls were used, and eight cones were positioned to define the change-of-direction course. Participants dribbled a ball through the course following the predefined path as quickly as possible. Each player performed three maximal trials, and the fastest time was used for analysis. Electronic timing gates (Witty, Microgate, Bolzano, Italy) were positioned at the start and finish lines to ensure precise time measurement, consistent with recommended sprint and agility testing procedures (35). All distances were measured with a measuring tape to ensure consistent course dimensions across testing sessions.

### 2.12 Statistical analysis

Descriptive statistics were calculated for all variables and reported as means, standard deviations (SD), and ranges. Normality was evaluated using a combination of Shapiro–Wilk tests and visual diagnostics (histograms and Q–Q plots). Pairwise associations between variables were examined using Pearson's correlation coefficient (*r*) when both variables were normally distributed, and Spearman's rank correlation coefficient (*ρ*) when normality was not met. For each correlation, the coefficient, *p*-value, 95% confidence interval (CI), and standard error (SE) are reported. Correlation analyses were considered exploratory in nature, and *p*-values were adjusted using the Holm procedure to account for multiple testing. Correlation strength was interpreted using conventional thresholds: 0.01–0.29 as small, 0.30–0.49 as moderate, and ≥0.50 as large (39). Talent rank was treated as the independent grouping variable. One-way ANOVA was used to examine differences between ranking groups in body composition (Fat% and FFMI) and physical performance (CMJ, ICODT-BALL, 40-m sprint and IR1). Effect sizes for ANOVA are expressed as eta-squared (*η*^2^), and *post-hoc* pairwise comparisons were adjusted using Holm correction. Standardised mean differences (Cohen's *d*) with 95% confidence intervals are also reported. Statistical analyses were performed using R version 4.3.2 (R Core Team, 2023) within RStudio (version 2025.09.2 + 418), with statistical significance set at *p* < 0.05.

## 3 Results

### 3.1 Descriptive data

A total of 88 female soccer players met the inclusion criteria and were included in the final analysis. The players had a mean chronological age of 11.9 ± 0.3 years and mean BA of 11.8 ± 1.1 years. Full descriptive values are presented in Table 1.

**Table 1:** Descriptive characteristics of 12-year-old female soccer players (*N* = 88).

Variable	Mean	SD	Min	Max
Descriptive				
Chronological age (years)	11.9	0.3	11.4	12.4
Height (cm)	154.3	7.2	136.0	169.8
Weight (kg)	44.4	8.4	28.8	72.0
Age when started soccer (years)	5.7	1.9	4.0	11.0
BMI	18.5	2.6	14.9	27.1
Anthropometric measurements				
BA (years)	11.8	1.1	8.8	14.6
Fat%	27.7	5.9	15.2	44.8
FFMI (kg/m^2^)	13.5	1.3	10.9	16.9
Performance variables				
ICODT-BALL (s)	27.6	3.1	19.8	36.2
40-m sprint (s)	6.9	0.5	6.1	8.5
CMJ (cm)	19.4	3.7	11.9	29.5
IR1 (m)	345.5	167.4	80.0	840.0

Values are presented as mean, SD, and range with min and max. BMI, body mass index; BA, bone age; Fat%, body fat percentage; FFMI, fat-free mass index; ICODT-BALL, Illinois change-of-direction test with ball; CMJ, countermovement jump; IR1, yo-yo intermittent recovery test level 1.

### 3.2 Correlations among maturity, body composition and physical performance

Correlations among physical performance, maturity and body composition variables are shown in Table 2. BA was not significantly correlated with performance, nor with Fat%. However, BA was positively correlated with FFMI (*r* = 0.64, *p*adj < 0.001). Fat% was positively correlated with 40-m sprint time (*ρ* = 0.60, *p*adj < 0.001) and ICODT-BALL performance (*ρ* = 0.42, *p*adj < 0.001) and negatively correlated with CMJ height (*ρ* = −0.64, *p*adj < 0.001).

**Table 2:** Correlations among performance, maturity and body composition variables (*N* = 88).

Variable	40-m sprint (s)	ICODT-BALL (s)	CMJ (cm)	IR1 (m)	BA (years)	Fat%
40-m sprint (s)						
ICODT-BALL (s)	0.54***					
CMJ (cm)	−0.72***	−0.44***				
IR1 (m)	0.13	−0.04	0.04			
BA (years)	−0.26	−0.11	−0.01	−0.11		
Fat%	0.60***	0.42***	−0.64***	−0.03	0.03	
FFMI (kg/m^2^)	−0.31	−0.19	0.11	−0.01	0.64***	0.11

Values are presented as Pearson's *r* or Spearman's *ρ*, as appropriate. Spearman's *ρ* was used when IR1 test performance or Fat% was included in the correlation. ICODT-BALL, Illinois change-of-direction test with ball; CMJ, countermovement jump; IR1, yo-yo intermittent recovery test level 1; BA, bone age; Fat%, body fat percentage; FFMI, fat-free mass index. Statistical significance is based on Holm-adjusted *p*-values across all pairwise correlations and is indicated as ***padj < 0.001.

### 3.3 Body composition and bone age between ranking groups

Comparing body composition with performance rank, a significant difference in Fat% (*p* = 0.015) was found between the three ranking groups. Higher ranked players (Rank 1) had the lowest Fat% (25.76 ± 5.52%) compared with those in Rank 2 (29.14 ± 5.77%) and Rank 3 (29.52 ± 5.70%). No significant difference was found between ranking groups in FFMI or BA. Ranking comparisons of body composition and BA are presented in Table 3.

**Table 3:** Anthropometric and body composition variables across ranking groups.

Variable	Rank 1 (*n* = 40)	Rank 2 (*n* = 26)	Rank 3 (*n* = 22)	df	*F*	*p*	*η* ^2^	*Post-hoc*	Cohen's *d* (95% CI)
BA (years)	11.81 ± 1.12 (9.36–13.84)	11.88 ± 0.97 (9.63–14.10)	11.57 ± 1.23 (8.80–14.57)	2.85	0.53	0.590	0.012	–	–
Fat%	25.76 ± 5.52 (15.18–39.86)	29.14 ± 5.77 (19.17–44.81)	29.52 ± 5.70 (20.10–42.50)	2.85	4.36	0.015	0.093	1 < 2	1 vs. 2: *d* = −0.60 [−1.12, −0.09]
								1 < 3	1 vs. 3: *d* = −0.67 [−1.22, −0.13]
FFMI (kg/m^2^)	13.74 ± 1.05 (11.79–15.78)	13.47 ± 1.25 (11.69–16.98)	12.98 ± 1.53 (10.91–16.43)	2.85	2.60	0.080	0.058	–	–

Values are presented as mean ± SD (min–max). BA, bone age; Fat%, body fat percentage; FFMI, fat-free mass index; df, degrees of freedom; *η*^2^, eta-squared effect size; Cohen's *d*, standardised mean difference.

### 3.4 Physical performance between ranking groups

Physical performance variables were compared across ranking groups. Significant differences in CMJ, ICODT-BALL, and 40-m sprint performance (*p* < 0.001) were observed between talent ranks, where Rank 1 significantly outperformed Ranks 2 and 3. The largest effect size for differences between ranking groups was observed for ICODT-BALL (*η*^2^ = 0.548), followed by 40-m sprint performance (*η*^2^ = 0.230) and CMJ height (*η*^2^ = 0.202). No significant differences were observed between ranking groups for IR1 test performance (*p* = 0.360). Ranking comparisons of physical performance are presented in Table 4.

**Table 4:** Physical performance variables across ranking groups.

Variable	Rank 1 (*n* = 40)	Rank 2 (*n* = 26)	Rank 3 (*n* = 22)	df	*F*	*p*	*η* ^2^	*Post-hoc*	Cohen's *d* (95% CI)
CMJ (cm)	21.20 ± 3.40 (13.70–29.50)	17.84 ± 3.28 (12.60–26.00)	17.92 ± 3.34 (11.90–23.10)	2.85	10.75	<0.001	0.202	1 > 2	1 vs. 2: *d* = 1.00 [0.47, 1.53]
								1 > 3	1 vs. 3: *d* = 0.97 [0.41, 1.53]
ICODT-BALL (s)	25.21 ± 1.97 (19.80–29.43)	28.56 ± 1.91 (26.19–33.62)	30.64 ± 2.52 (25.15–36.20)	2.85	51.57	<0.001	0.548	1 < 2	1 vs. 2: *d* = −1.72 [−2.31, −1.13]
								1 < 3	1 vs. 3: *d* = −2.50 [−3.19, −1.80]
								2 < 3	2 vs. 3: *d* = −0.94 [−1.56, −0.33]
40-m sprint (s)	6.76 ± 0.37 (6.10–7.72)	7.08 ± 0.44 (6.27–8.48)	7.28 ± 0.44 (6.41–8.26)	2.85	12.67	<0.001	0.230	1 < 2	1 vs. 2: *d* = −0.81 [−1.33, −0.29]
								1 < 3	1 vs. 3: *d* = −1.32 [−1.91, −0.74]
IR1 (m)	359.00 ± 163.67 (80–720)	306.15 ± 150.07 (80–680)	367.27 ± 192.28 (120–840)	2.85	1.03	0.360	0.024	–	–

Values are presented as mean ± SD (min–max). CMJ, countermovement jump; ICODT-BALL, Illinois change-of-direction test with ball; IR1, yo-yo intermittent recovery test level 1; df, degrees of freedom; *η*^2^, eta-squared effect size; Cohen's *d*, standardised mean difference.

## 4 Discussion

This study examined differences in bone age (BA), body composition, and physical performance across ranking groups in 12-year-old female soccer players. The main findings were that higher-ranked players demonstrated superior ICODT-BALL (agility with the ball) performance, 40-m sprint performance and CMJ height, as well as lower Fat%, whereas no differences were observed between ranking groups in BA, FFMI, or IR1 test performance.

The present findings demonstrate that sprint speed, change-of-direction ability, and jump performance were the primary factors differentiating ranking groups. This is consistent with previous research in female youth soccer showing that faster sprint times, greater explosive power, and superior agility characterise players who progress within elite pathways (6, 14, 15). Consistent with the present findings, linear sprint performance is strongly associated with change-of-direction ability and competitive level across youth soccer populations (35). The particularly large effect observed for ICODT-BALL likely reflects the combined physical and technical demands of changing direction while controlling the ball. Inclusion of the ball increases coordination and perceptual-motor demands and makes the task more representative of match play (40). This may explain why agility tests incorporating ball control appear to differentiate competitive level more effectively than non-ball change-of-direction tasks in female youth soccer (15). Nevertheless, ICODT-BALL should be interpreted as a soccer-specific composite performance measure rather than as an isolated measure of physical capacity, as performance depends on both change-of-direction ability and ball-control skill. The strong associations observed between ICODT-BALL, sprint performance and CMJ height further suggest that speed and lower-limb power are closely related to dribbling-based agility in this age group (35). Longitudinal studies in female academy players show that physical performance improves with maturation, but development is non-linear and strongly influenced by body composition (41). Given that those studies relied on predicted rather than direct maturity measures, the present findings using direct BA assessment further support the view that body composition exerts a more consistent influence on performance development in girls.

Body composition differed between ranking groups, with higher-ranked players exhibiting lower Fat% than lower-ranked peers. However, these differences were small relative to the pronounced separation observed in ICODT-BALL performance, sprint time, and jump height, indicating that body composition likely serves as a secondary rather than a primary discriminator in ranking (41, 42). Higher Fat% was associated with slower sprint times, poorer ICODT-BALL performance and lower CMJ height, suggesting that adiposity may be linked to less favourable speed- and power-related performance in early adolescent players. This aligns with evidence suggesting that pubertal increases in fat mass in girls may reduce the extent to which growth-related gains in body size translate into improved physical performance (11, 20).

No differences in BA were observed between ranking groups, suggesting that skeletal maturity was not strongly associated with coach ranking in this group. Although the sample was restricted to one birth year, BA still showed meaningful variation, suggesting that the absence of ranking-group differences was not solely attributable to restricted maturity variation. Although BA showed a small negative correlation with 40-m sprint time, this association did not remain statistically significant after Holm adjustment. In contrast, BA was positively correlated with FFMI, suggesting that skeletal maturity is more closely related to fat-free mass relative to height than to physical performance in this age-specific cohort. Notably, BA was not significantly correlated with Fat%, indicating that although maturation and lean mass develop in parallel, the accumulation of fat mass, which was most strongly associated with performance in this cohort, appears to be relatively independent of skeletal maturity at this age. This aligns with evidence from female youth athletes showing that maturity-related effects on speed and power are smaller, more variable, and less consistently expressed than in boys (21). Although the sample was relatively homogeneous in chronological age, sex and sporting context, it still showed meaningful variation in BA and body composition. Therefore, the weak maturity-related differences observed in the present study should not be attributed solely to sample homogeneity, but rather as reflecting both the age-specific sampling frame and the less consistent maturity–performance associations reported in girls. Collectively, the present findings support the view that functional performance, rather than biological maturity, is most strongly associated with performance differentiation in early adolescent female soccer (13, 21).

IR1 test performance did not differ significantly between ranking groups and was not correlated with BA, Fat% or FFMI. Field-based endurance tests are susceptible to pacing behaviour, perceived exertion and fatigue tolerance, particularly in youth populations (33, 43), which may partly explain the IR1test's limited discriminatory ability, rather than reflecting a true absence of endurance differences between groups. Nevertheless, these findings suggest that intermittent endurance is not a primary consideration in coach ranking at this age. The IR1 test may therefore hold greater value as a longitudinal monitoring tool, tracking endurance development as match demands increase across adolescence (33, 44).

### 4.1 Strengths and limitations

A key strength of this study is the use of validated objective methods to assess BA, body composition, and physical performance. BA assessed using x-ray is considered a gold-standard method for evaluating biological maturation in youth athletes (24, 25). Body composition measured using DXA scan is widely recommended as a precise technique for assessing fat mass and fat-free mass in athletic populations (26, 45). However, several study limitations should be acknowledged. The cross-sectional limits causal inference and does not allow examination of developmental changes over time. Longitudinal research is therefore required to clarify how relationships between BA, body composition, physical performance, and ranking evolve throughout adolescence. Sprint and endurance tests were conducted outdoors, which may have introduced environmental variability despite applying standardised testing procedures. In addition, although the IR1 test is soccer-specific and widely used, it does not represent a direct measure of aerobic capacity and may be influenced by pacing strategy, motivation, and neuromuscular fatigue in young athletes (33, 34). The study did not include direct assessments of maximal strength, which may have provided additional insight into sprint and jump performance (9, 46). Although players were recruited from multiple teams, exploratory analyses did not reveal systematic differences between teams, suggesting that observed variability in performance was primarily driven by individual developmental differences rather than by team-specific training practices. Finally, the ranking variable was based on subjective coach evaluation rather than standardised criteria. Coaches' assessments may be influenced by individual experience, perceptual bias, situational factors, unmeasured technical or tactical qualities not captured by the objective measures administered in this study. Accordingly, ranking should be interpreted as an indicator of observed performance at age 12. Although several studies in youth and adolescent soccer report associations between intermittent endurance and competitive level or match performance (34, 41, 47), this likely reflects age-related shifts in performance determinants, with endurance increasing in importance across maturation while speed and power dominate in early adolescence (35, 41). Match context may further explain the limited relevance of the IR1 test in the present cohort, as players at this age compete in small-sided formats that place greater demands on repeated acceleration than on sustained aerobic running. The present study used a complete-case analytic approach, whereby 59 of the 147 Wave 1 participants were excluded from the final analysis due to missing data attributable primarily to technical failure or incomplete data capture during specific testing batteries. A formal statistical comparison of included and excluded participants was not conducted, however, as exclusions were driven by technical data loss rather than voluntary dropout, systematic selection bias related to player characteristics is considered unlikely, though cannot be entirely ruled out.

## 5 Conclusions

Performance-based ranking of 12-year-old Icelandic female soccer players by their coaches was associated with speed and power-related outcomes (ICODT-BALL, 40-m sprint, and CMJ height) and Fat%. However, no differences were observed in BA, FFMI or IR1 test performance between ranking groups. Instead, ranking appeared to reflect current speed- and power-related performance characteristics more than BA. From an applied perspective, objective performance testing may help describe the physical performance characteristics associated with coach ranking at this age. These findings suggest that functional physical performance, particularly ball-related change-of-direction ability and sprint speed, may be more closely associated with coach ranking at this age than BA. However, longitudinal and intervention studies are needed to determine whether targeted training of these qualities influences future player development.

## Data Availability

The raw data supporting the conclusions of this article will be made available by the authors, without undue reservation.
